# TIMP-1 is a novel serum biomarker for the diagnosis of colorectal cancer: A meta-analysis

**DOI:** 10.1371/journal.pone.0207039

**Published:** 2018-11-20

**Authors:** Chunyan Meng, Xiaowei Yin, Jingting Liu, Kaifeng Tang, Hongchao Tang, Jianhua Liao

**Affiliations:** 1 Department of General Surgery, Zhejiang Hospital, Hangzhou, Zhejiang, China; 2 Department of General Surgery, People’s Hospital of Anji, Huzhou, Zhejiang, China; 3 Department of Emergency, Sir Run Run Shaw Hospital, Zhejiang University School of Medicine, Hangzhou, Zhejiang, China; University of New Mexico, UNITED STATES

## Abstract

**Purpose:**

Tissue inhibitor of metalloproteinase-1 (TIMP-1) is a glycoprotein involved in cell survival and tumorigenesis. There have been some promising results regarding the diagnostic value of TIMP-1 for patients with colorectal cancer (CRC). The aim of the present study was to assess the diagnostic accuracy and clinical utility of serum TIMP-1 in CRC patients through meta-analysis.

**Methods:**

A systematic search of online databases was performed to collect eligible studies. The pooled sensitivity, specificity, diagnostic odds ratio (DOR), and summary receiver operator characteristic (SROC) curve were generated from accuracy data using the random-effects model. Fagan’s nomogram and the likelihood matrix were applied to estimate the clinical utility of TIMP-1.

**Results:**

A total of 9 eligible studies with 1886 patients were included. Among the patients, 819 were pathologically diagnosed with CRC, whereas 1067 did not have adenomas or other cancers. The overall sensitivity, specificity, and DOR of TIMP-1 for the diagnosis of CRC were 0.65 (95% confidence interval (CI): 0.57–0.72), 0.87 (95% CI: 0.76–0.94), and 12.73 (95% CI 5.71–28.38), respectively. The area under the SROC was 0.77 (95% CI, 0.73–0.81), suggesting the potential diagnostic value of TIMP-1 in CRC patients. Among patients with a pretest CRC probability of 20%, posttest probabilities were 56% and 9% for positive and negative TIMP-1 results, respectively.

**Conclusions:**

TIMP-1 expression exhibits an upper moderate diagnostic value in CRC, and TIMP-1 assessment may be useful as a noninvasive screening tool for CRC in clinical practice.

## Introduction

Cancer is the leading cause of death worldwide, and much of this increasing burden is due to the growth and aging of the population, with a particular association with unwholesome lifestyle behaviors. Increased awareness of the importance of early cancer detection among the medical community has occurred in recent years, with a greater understanding of the association between patient prognosis, clinicopathological factors, imaging detection, and biomarkers [1–2]. Colorectal cancer (CRC) is the third most prevalent cancer in males and the second most prevalent cancer in females, with an estimated 1.4 million cases and 693,900 deaths per year. CRC occurrence has a certain geographical distribution, with the highest incidences found in Australia/New Zealand, Europe, and Northern America. Despite decreasing CRC mortality rates in a large number of countries worldwide, increasing mortality is still occurring in countries that have insufficient resources and growing incidence, notably in Western Asia and Eastern Europe [3]. As with other cancers, optimal treatment depends on accurate diagnosis, and early detection is clearly a key factor in reducing mortality among CRC patients [4]. Despite rapid advances in CRC screening, including colonoscopy, fecal occult blood testing (FOBT), immunochemical FOBT (iFOBT), and fecal DNA analysis, which are considered the most reliable and pervasive tests, timely and early detection of tumors has not improved satisfactorily. Furthermore, excessive complications, high costs, and lack of compliance are continually reducing the applicability and sensitivity of testing. In addition to genetic predisposition, carcinoembryonic antigen (CEA), C-reactive protein, serum CD26, and other biomarkers have recently been demonstrated to have the potential to complement CRC screening methods [5–7]. Regardless, none of these markers has the ability to explain all individual differences in CRC detection. Because there are no biomarkers that demonstrate both high sensitivity and high specificity for CRC, new tools improving the detection rate of CRC screening are needed. Although researchers have confirmed that iFOBT has adequate sensitivity and specificity (sensitivity of 65.8% at 95% specificity) [8], approaches to increase compliance for CRC detection still need to be taken into consideration.

Tissue inhibitors of matrix metalloproteinases (TIMPs), naturally occurring tissue inhibitors of matrix metalloproteinases (MMPs), partly regulate the proteolytic activity of MMPs, stimulating tumor growth and inhibiting tumor cell apoptosis, and also act as a functional regulator of malignant transformation [9]. In addition, some literature reports that imbalance between MMPs and TIMPs is a risk factor in tumorigenesis [10]. At present, there are four recognized types of TIMPs: TIMP-1, TIMP-2, TIMP-3 and TIMP-4. The main functional TIMP is TIMP-1, which is encoded by a gene located on chromosome Xp11.23–11.4, and it is primarily found in the intercellular matrix and plasma [11–13]. Upregulated expression of TIMP-1 is observed in various tumor tissues and is a significant indicator of cancer invasion, metastasis and survival of patients with cancers [14–16]. In addition to its crosstalk with MMPs, many convincing studies report that TIMP-1 can regulate apoptosis and proliferation in an MMP-independent manner and play a role in colorectal carcinogenesis [17]. In particular, many studies demonstrate that TIMP-1 can be used as a biomarker for prognosis in CRC patients and as a diagnostic marker for detecting CRC [1, 18]. To date, there is unambiguous evidence for the diagnostic and prognostic value of blood levels of TIMP-1 in CRC, and a few studies have employed meta-analysis to assess the overall value of TIMP-1 in prognosis [19]. However, there is no systematic analysis of the diagnostic value of TIMP-1 in CRC detection. Therefore, assessment of its ability to facilitate tumor diagnosis is imperative. In this study, we conducted a meta-analysis based on a comprehensive search of the relevant literature to evaluate the overall diagnostic value of TIMP-1 in CRC.

## Materials and methods

### Data sources and search strategy

On April 8, 2018, we systematically searched the PubMed, PMC, Springer, Science Direct, Wiley Online Library, Web of Knowledge (ISI) and Web of Science using the search terms “(TIMP-1 or Tissue inhibitor of metalloproteinase-1) and (colorectal or colon and rectal) and (cancer or carcinoma or tumor or neoplasm) and (serum or sera or serums or blood or plasma) and (diagnosis or sensitivity or specificity or ROC or AUC)”, without language restriction. References, relevant systematic reviews and meta-analyses were also checked to prevent missed search results. This meta-analysis was performed in accordance with the reporting checklist and flow diagram of the Quality of Reporting of Meta-analysis (QUOROM) [20].

### Inclusion and exclusion criteria

The inclusion criteria for literature were as follows: (1) patients were pathologically diagnosed with CRC, and the matched control individuals were without colorectal adenoma or any type of cancer; (2) TIMP-1 protein levels in blood samples was examined; (3) all blood samples from CRC patients were drawn preoperatively; (4) the endpoint was set as CRC patients versus matched control individuals; (5) the studies provided data for sensitivity, specificity, or the receiver operating characteristic (ROC) curve and the area under the curve (AUC) and sufficiently constructed 2*2 contingency tables; (6) the studies involved more than 20 cases; and (7) retrospective or prospective observational studies were reported. Animal experiments, letters, editorials, meeting abstracts, case reports, reviews, meta-analysis and conferences were excluded.

### Data extraction and quality assessment

Data regarding baseline characteristics (e.g., first author, publication date, country, sample size or types, mean or median age, recruitment time, assay type) and diagnostic results (e.g., cutoff value, sensitivity, specificity, AUC) were extracted from each study. Two investigators independently extracted the data to obtain information and were able to conduct reasonable discussions in accordance with pre-specified rules or existing science when they encountered inconsistencies. In addition, the quality of each eligible study was assessed by Quality Assessment of Diagnostic Accuracy Studies 2 (QUADAS-2), which offers a considerably improved tool for distinguishing between bias and applicability [21]. Briefly, we considered judgment of each study based on 4 key domains (patient selection, index test, reference standard, and flow and timing) to be appropriate.

### Statistical analysis

The software STATA 14.0 (Stata Corporation, College Station, TX, USA) and Review Manager 5.2 (The Cochrane Collaboration, NCC, CPH, Denmark) were utilized to perform the meta-analysis. The pooled sensitivity, specificity, positive likelihood ratio (LRP), negative likelihood ratio (LRN), diagnostic odds ratio (DOR), and summary receiver operator characteristic (SROC) curve were calculated from accuracy data, and the corresponding 95% confidence intervals (CI) were further obtained if necessary. AUC was used to represent the diagnostic accuracy of TIMP-1 measurement, besides, the AUC is between 0 and 1, and AUC = 1 means the prediction model is perfect. Fagan’s nomogram and the likelihood matrix were used to evaluate the clinical utility of TIMP-1 measurement. Moreover, Spearman correlation analysis was performed to reveal the presence of the threshold effect. The Cochran-Q method and inconsistency index (I^2^) were adopted to investigate and quantify heterogeneity among the studies. The pooled estimation was evaluated by the fixed-effect model only if the Cochran-Q method of P > 0.10 or I^2^ < 50% was met. Otherwise, the random-effect model was applied. Moreover, we performed meta-regression and subgroup analysis to estimate the source of heterogeneity caused by the nonthreshold effect. Simultaneously, we also applied Deeks’ test to investigate publication bias. Pooled accuracy data are presented as forest plots. Two-sided tests were used in all the analyses. P<0.05 was considered statistically significant.

## Results

### Characteristics of the included studies

**Fig 1:**
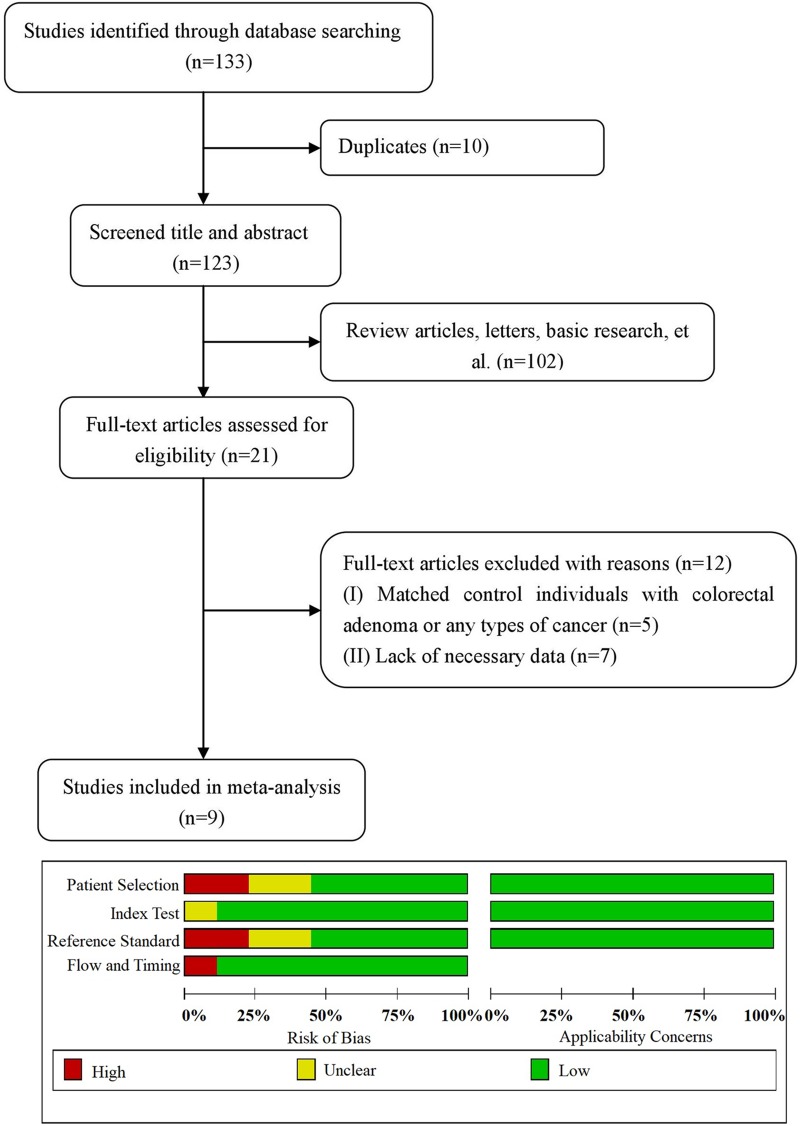
Flow diagram for the study selection process and quality assessment using QUADAS-2. The quality of each eligible study was assessed by QUADAS-2. It summarized ‘‘risk of bias” and ‘‘applicability concerns” through judging each domain for each included study.

The preliminary literature search of the selected electronic databases generated 133 hits. After carefully screening the titles and abstracts of each article, 10 were further excluded because they were duplicates; 102 reports were also excluded because they were, for example, review articles, letters, or basic research. In total, 21 articles were fully assessed for eligibility. Of these, 5 were excluded because they involved subsets of patients with other diseases (e.g., colorectal adenomas and other cancers), and 7 were excluded due to a lack of necessary data. Therefore, 9 eligible studies published from 2002 to 2014 were ultimately included. The precise selection process of the literature search is shown in Fig 1, and the detailed information is summarized in Table 1. A total of 1886 patients from China [22], Denmark [17, 23–24], Germany [25–26], the Netherlands [27], and Poland [18, 28] were included in this meta-analysis; 819 were pathologically diagnosed with CRC, and 1067 did not have colorectal adenomas or other cancers. Five were prospective studies, and 4 were retrospective. Patient recruitment time was well defined in five studies. Sensitivity and specificity were obtained through the ROC curve or directly through the published data. One study reported an indeterminate value for the AUC because two groups had been stratified to assess the diagnostic value but only one AUC was given. Four studies provided the cutoff value, from which the corresponding sensitivity and specificity were obtained. In all 9 studies, TIMP-1 expression was evaluated by enzyme-linked immunosorbent assay (ELISA). For the included studies, “patient selection” and “reference standard” revealed certain shortcomings (44.4% (4/9) and 44.4% (4/9), respectively), which may indicate bias regarding inclusion. In other words, three studies were judged as “low” in all domains related to bias and applicability, and the other included studies had an overall judgment of “at risk of bias”.

**Table 1:** Main characteristics of studies included in the meta-analysis.

Author(Year)	Study design	Country	Patients/controls	age (years)	Recruitment time	Assay type	Cutoff value	Sensitivity	Specificity	AUC	QUADAS-2 assessment ^g^
Niewiarowska K(2014)	retrospective	Poland	43/24	63^d^	NS	ELISA	537.8 ng/ml	0.674	0.667	0.666	At risk/ low concern
Holten-andersen L(2012)	prospective	Denmark	56/105	61^e^	2006.8–2007.1	ELISA	NS	0.52^f^	0.78^f^	0.68	Low risk/ low concern
Tao S(2012)	prospective	Germany	67/217	NS	2005–2008	ELISA	NS	0.52^f^	0.60^f^	0.58	Low risk/ low concern
Yuan C B(2010)	retrospective	China	40/40	66^e^	2008.3–2009.3	ELISA	224.9 ng/ml	0.70	0.60	0.727^f^	At risk/ low concern
Mroczko B(2010)	retrospective	Poland	75/70	20–78	2003.9–2006.5	ELISA	203 ng/mL	0.85^f^	0.74^f^	0.832	Low risk / low concern
Karl J (2008)	prospective	Germany^a^	101 ^b^ /252	64.5^d^	NS	ELISA	NS	0.73	0.95	UC	At risk/ low concern
Karl J (2008)	prospective	Germany^a^	85 ^c^ /252	63.3^d^	NS	ELISA	NS	0.72	0.95	UC	At risk/ low concern
Sørensen N M (2008)	prospective	Denmark^a^	30/180	NS	NS	ELISA	NS	0.60	0.98	0.88	At risk/ low concern
Waas E T(2005)	prospective	Netherland	91/51	28–86	The median follow-up was 27.5 months	ELISA	NS	0.56	0.95	0.81	At risk/ low concern
Holten-andersen M N(2002)	retrospective	Denmark	588/108	33–90	NS	ELISA	376 ng/mL	0.55	0.95	0.87	At risk/ low concern

^a^Country of most patients

^b^Patients without FOBT testing or visible blood in stool

^c^Patients without restrictions applied

^d^Mean age

^e^Median age

^f^Data obtained through the ROC curve

^g^overall Judgment in “risk of bias” and “applicability concerns” by QUADAS-2

NS:Data were not shown

UC:Data were unclear.

### Assessment of publication bias and heterogeneity

**Fig 2:**
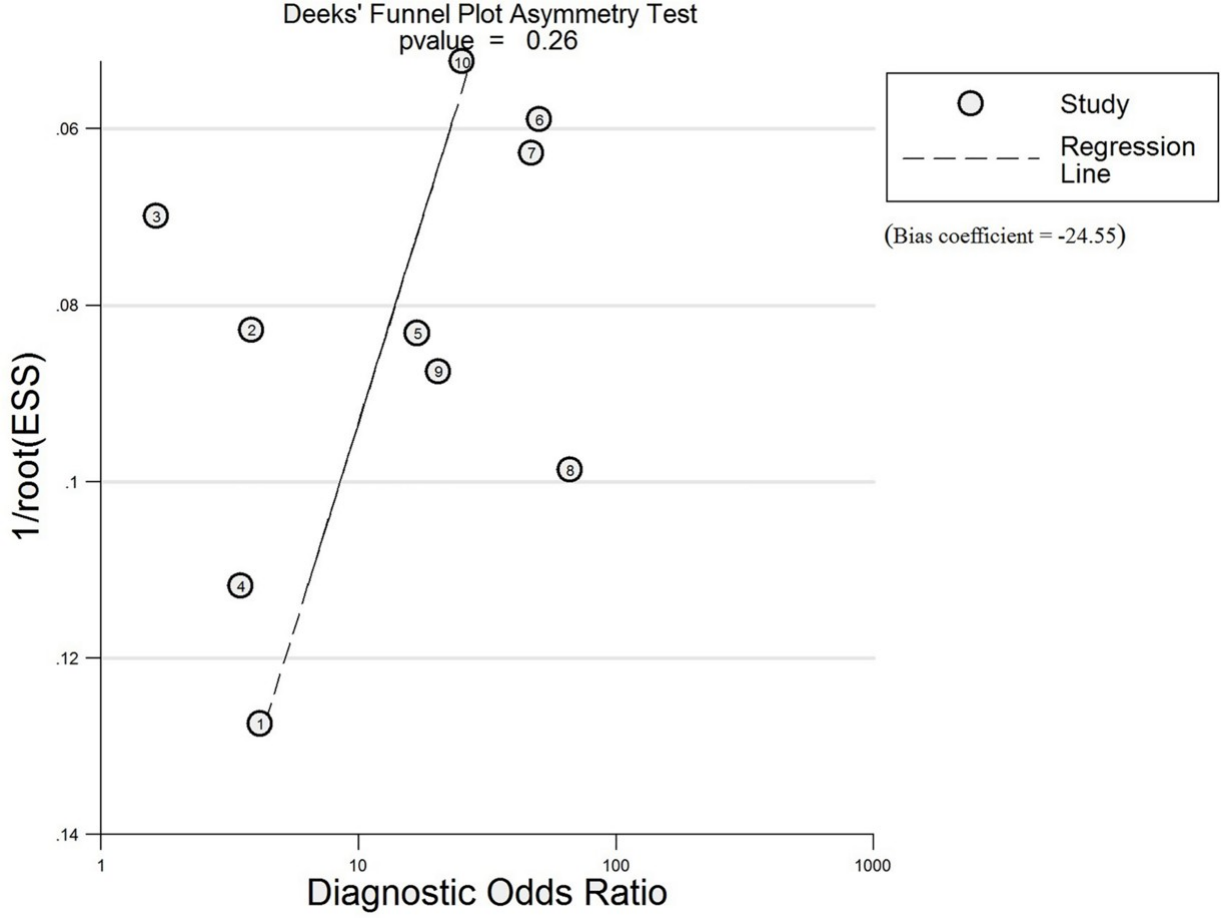
Results of Deeks’ funnel plot asymmetry test for the evaluation of potential publication bias. Every point represents one study and the line is the regression line. The nonsignificant slope indicates that no significant bias was found. The p value is of 0.26 (Bias coefficient = -24.55).

**Fig 3:**
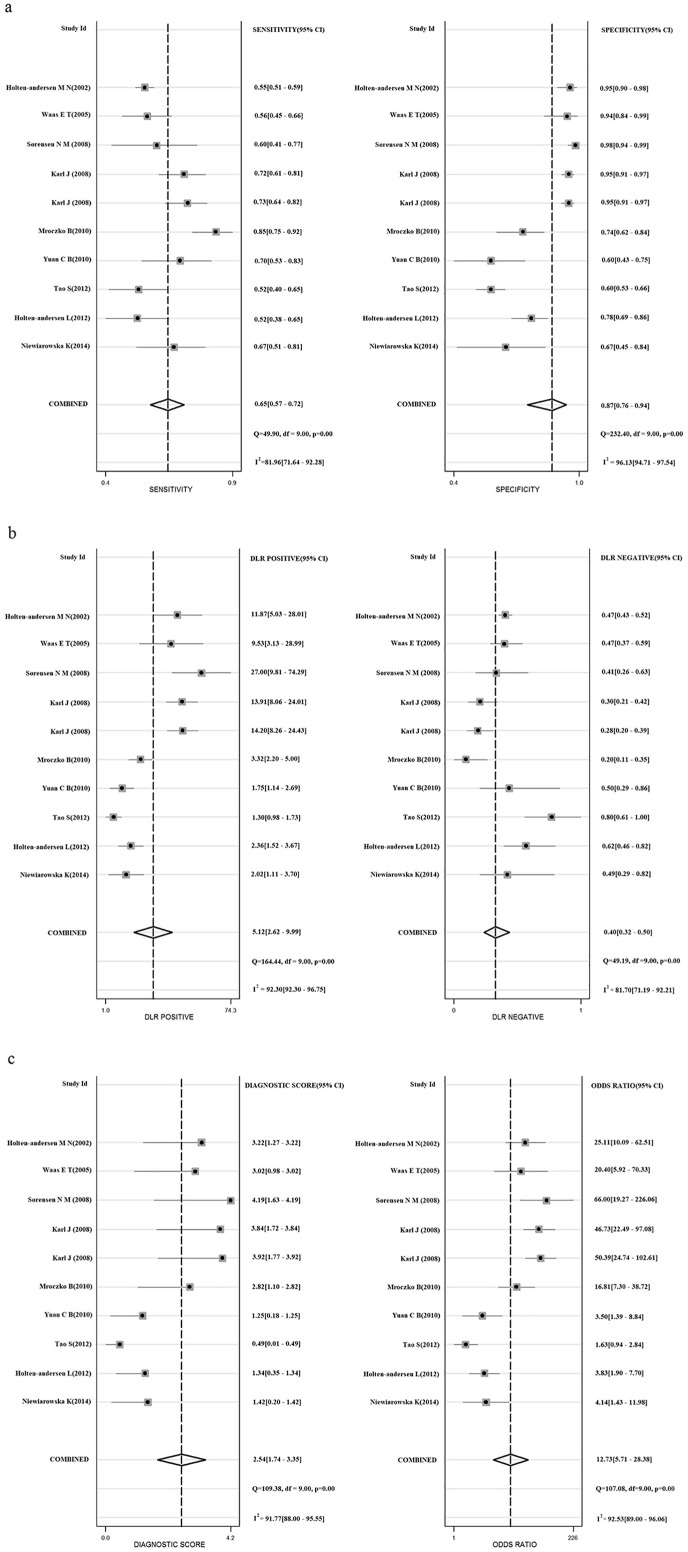
**Forest plots show the pooled sensitivity and specificity (a), the pooled positive likelihood ratio and negative likelihood ratio (b), the pooled diagnostic score and diagnostic odds ratio(c) for assessing the diagnostic value of TIMP-1in colorectal cancer.** The forest plots show the pooled diagnosis index of TIMP-1 for the diagnosis of CRC. The individual study symbol is shown as point and the pooling symbol is shown as point. Inconsistency is used to quantify the heterogeneity caused by nonthreshold effect. For these studies, DerSimonian–Laird was used to pool these data.

**Fig 4:**
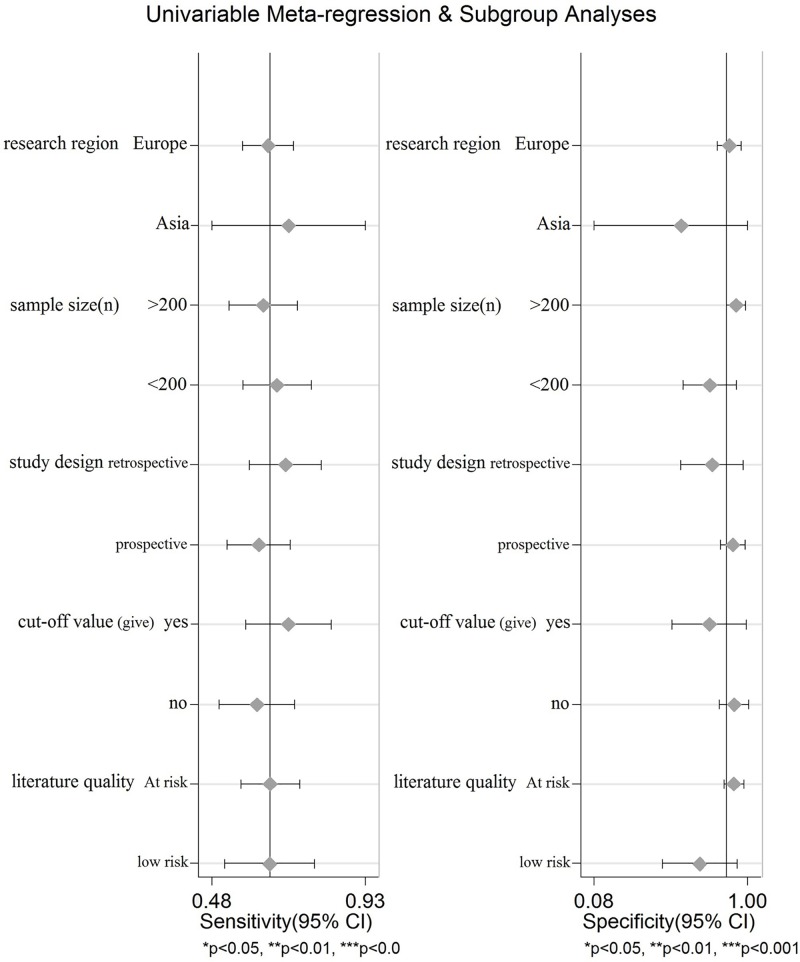
Meta-regression and subgroup analyses for potential sources of heterogeneity. The forest plots show the pooled diagnosis index of TIMP-1 for the diagnosis of CRC in specified covariates. The pooling symbol is shown as point. P-values of covariants in the meta-regression analysis are more than 0.05, which shows that none of the factor that is responsible for the heterogeneity.

For optimal diagnosis accuracy, we first calculated the bias coefficient and p value using Deeks’ test. The bias coefficient was -24.55, and the P value was 0.26, indicating no publication bias among eligible studies for TIMP-1 (Fig 2). Furthermore, heterogeneity among the included studies was measured using the Spearman test and Cochran-Q method. The Spearman correlation coefficient between the logit of the true positive rate and the logit of the false positive rate was -0.061 (p = 0.868), and the Cochran-Q value of the likelihood ratio test (LRT) was 69.81 (P<0.001). As shown in Fig 3A–3C, the I^2^ values of the pooled sensitivity analysis and DOR estimate were greater than 75%. The above results suggest the presence of a nonthreshold effect, with heterogeneity also present. Therefore, we used meta-regression and subgroup analysis to assess the potential source of the observed heterogeneity. However, univariate stratified analysis of the research region, sample size, study design, cut-off value and literature quality did not indicate the one factor that was responsible for the heterogeneity (Fig 4, P>0.05).

### Performance of TIMP-1 in diagnosing CRC

**Fig 5:**
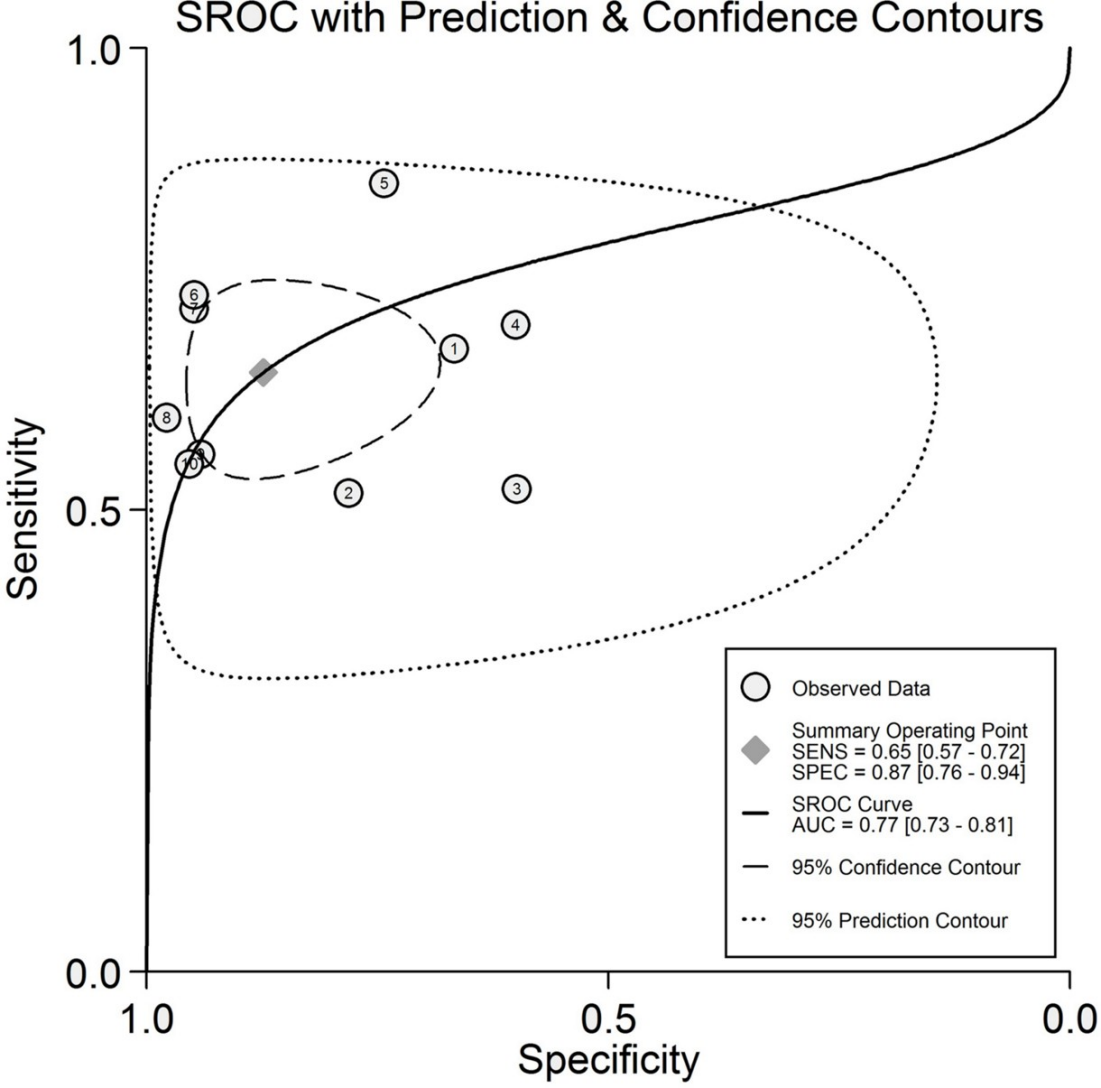
Summary ROC curve of the TIMP-1 diagnostic value in colorectal cancer. Every point represents an included study. The diamond shape represents the summary sensitivity and specificity. The AUC is 0.77, which implies an upper moderate diagnostic accuracy for diagnosing CRC.

The results of comprehensive analysis of the included studies are summarized in Fig 3A–3C. Due to the extreme heterogeneity, sensitivity, specificity, and other diagnostic values of TIMP-1 for diagnosing CRC were calculated using the DerSimonian–Laird method (random-effect model). The overall sensitivity and specificity of TIMP-1 for CRC diagnosis were 0.65 (95% CI: 0.57–0.72) and 0.87 (95% CI: 0.76–0.94), respectively. The pooled positive likelihood ratio and negative likelihood ratio were 5.12 (95% CI, 2.62–9.99) and 0.40 (95% CI, 0.32–0.50), respectively. We further obtained the diagnostic score (DS) and DOR to better illustrate the discriminant effect of TIMP-1 measurement, with 2.54 (95% CI 1.74–3.35) for the former and 12.73 (95% CI 5.71–28.38) for the latter, suggesting that TIMP-1 measurement is an effective diagnostic method. Moreover, the area under the SROC was 0.77 (95% CI, 0.73–0.81), which is consistent with upper moderate diagnostic accuracy (Fig 5) [29].

### Evaluation of clinical utility

**Fig 6:**
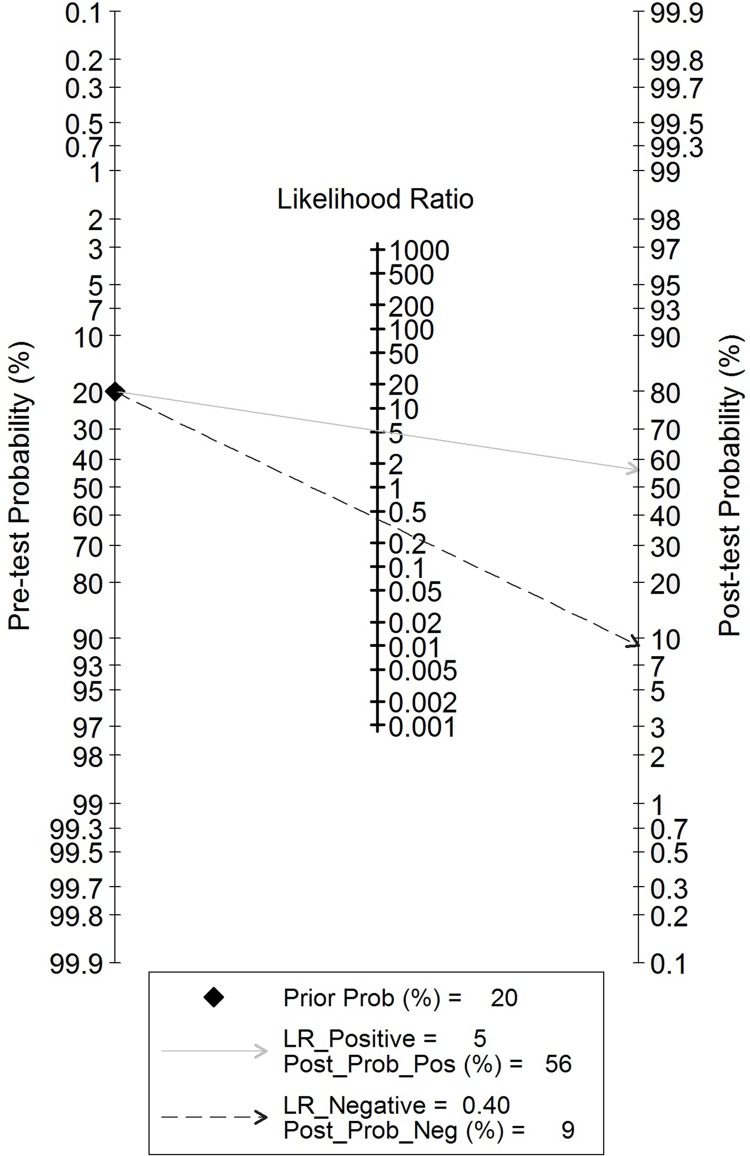
Fagan’s Nomogram for the elucidation of posttest probabilities. With a pretest probability of CRC of 20%, the posttest probabilities of CRC, given positive and negative TIMP-1 results, are 56% and 9%, respectively.

**Fig 7:**
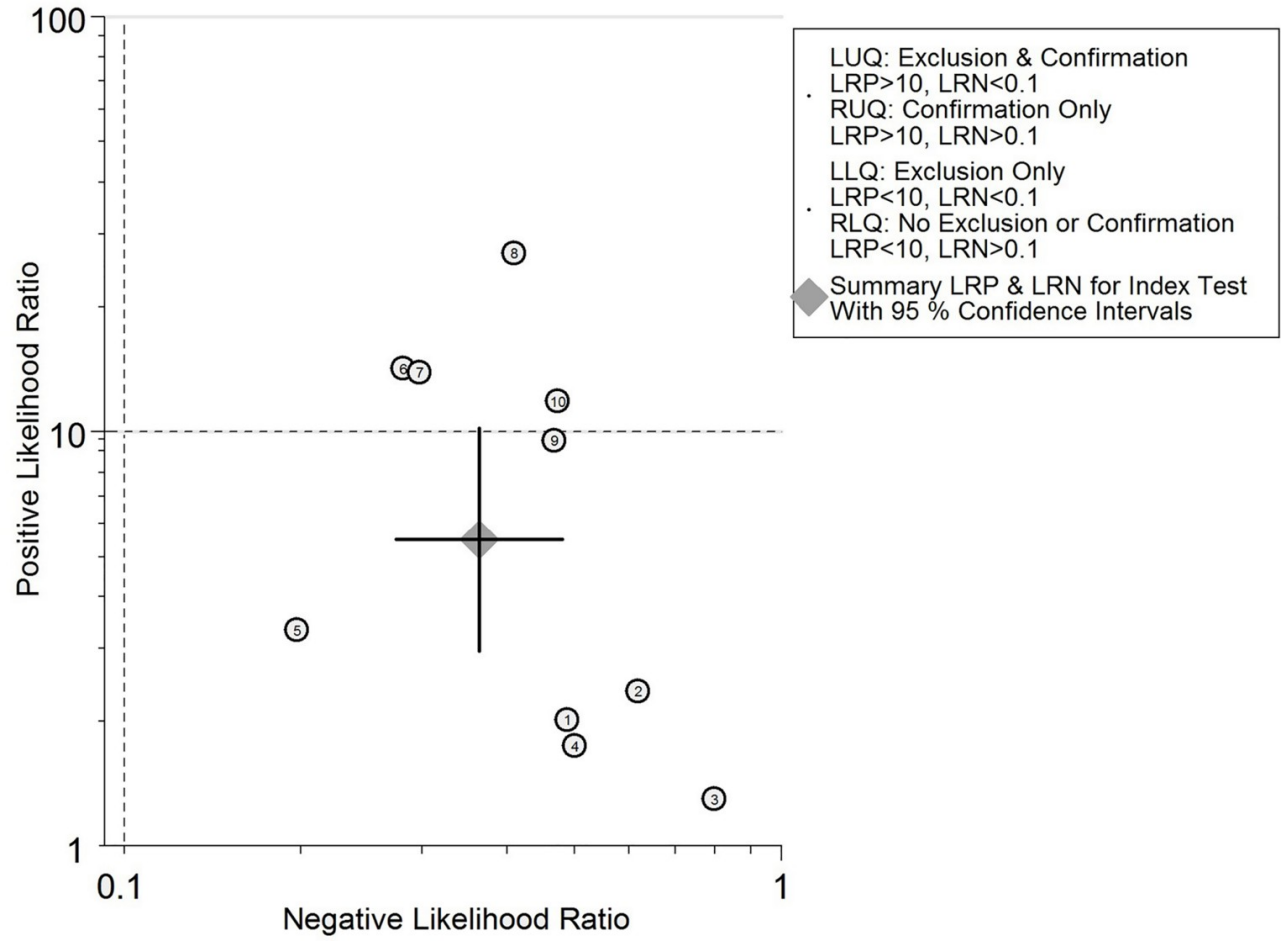
Likelihood matrix for the overall distribution of the studies. Each point corresponds to a study. None of the included studies were found on the bottom left side of the matrix, which shows that they report reasonable sensitivity.

To better elucidate the role of TIMP-1 in CRC screening, we utilized likelihood ratios to simulate a clinical scenario using a certain pretest probability of CRC. In detail, for those with a CRC pretest probability of 20%, if assessment of TIMP-1 for cancer detection was positive, the posttest probability of having CRC rose to 56%; the probability of having CRC was 9% with a negative TIMP-1 result, which may rule out CRC (Fig 6). In addition, the likelihood matrix was further employed to assist in describing how to use the diagnostic finding from the TIMP-1 assay to calculate the posttest probability of CRC, as illustrated in Fig 7. None of the included studies were found on the bottom left side of the matrix (LRP<10 and LRN<0.1), thus indicating significantly increased probability of a diagnosis of CRC.

## Discussion

An efficient predictive biomarker should benefit patients with early treatment, thereby avoiding the adverse effects of tumor progression. Overall, the prognosis of patients with CRC has improved with important discoveries regarding the ability of CEA, C-reactive protein, serum CD26 and TIMP-1 to assist in CRC diagnosis, in addition to innovative surgical techniques and adjuvant therapy [5–7]. Although pathologic diagnosis, colonoscopy, and FOBT remain the most reliable tests for detecting CRC, these tests require good patient compliance, are invasive and relatively expensive, and are easily influenced by other gastrointestinal and anorectal diseases [30]. Therefore, both the development of advanced treatment techniques and reliable biomarkers must be improved to detect tumors. As this meta-analysis reveals the importance of TIMP-1 expression in the detection of CRC, TIMP-1 assessment appears to be an effective screening tool for the diagnosis of CRC before it progresses to unresectable stages.

Synthesized in stromal cells to regulate proteinase reactions, TIMPs play a key role in CRC invasion and metastasis. TIMP-1, a 28-kDa glycoprotein released by endometrial cells, fibroblasts and cancer cells, cooperates with MMPs to form noncovalent 1:1 stoichiometric complexes and plays a pivotal role in tumorigenesis, progression and metastasis by inhibiting the matrix-degrading properties of endopeptidases or acts through FAK-PI3K/AKT and MAPK pathways; increased expression of TIMP-1 is observed in CRC tissues and patient serum [1, 31–33]. In addition to its function in inhibiting metalloproteinases, TIMP-1 was recently reported to regulate tetraspanin/integrin-mediated cell survival signal transduction pathways and to activate Ras via the TYK/MAPK pathway involved in cell death, proliferation, transformation, differentiation, and apoptosis [34–35]. Possible participation of TIMP-1 in angiogenesis was reported in breast carcinoma cells in vivo, and overexpression of VEGF may have an important role in this TIMP-1-mediated effect [36]. Furthermore, serum levels of TIMP-1 were evaluated in recent studies, revealing that TIMP-1 might predominate over the effects of MMPs in the very early stages of CRC, and the TIMP-1 protein have been linked to the degree of malignancy, particularly in colorectal carcinogenesis [1, 5, 37–38]. In view of these findings, studies have focused on the role of TIMP-1 in the diagnosis and prognosis of patients with CRC, and many researchers have published data on its diagnostic value in CRC [17–18, 22–28]. Thus, we focused on the relationship between TIMP-1 and CRC detection via meta-analysis.

We included 9 studies comprising 819 CRC patients and 1067 healthy controls in this meta-analysis. Significant diagnostic value of TIMP-1 expression in the blood of patients with CRC compared to the healthy control group was observed, and combined analysis showed that TIMP-1 had good performance characteristics, with upper moderate sensitivity and specificity and remarkable clinical utility. However, despite the fact that we incorporated the literature into combined analysis, substantial heterogeneity still existed. Heterogeneity is an important factor in the interpretation of meta-analyses, and to assess whether the observed heterogeneity was caused by the threshold effect, we performed the Spearman test. The value of -0.061 (P = 0.868) obtained indicated no threshold effect. We then used the Cochran-Q and I^2^ tests to further analyze and quantify heterogeneity and found strong heterogeneity, as evidenced by the Cochran-Q (P<0.001) and I^2^ (I^2^>75%) values. Hence, the source of heterogeneity present in this analysis on CRC detection was subsequently evaluated using meta-regression and subgroup analysis. The results revealed P values of more than 0.05 for all specified covariates, indicating that we failed to identify the sources of heterogeneity. Thus, a random-effect model was necessary for our further analysis to eliminate some heterogeneity. Interestingly, subgroup analysis by those specified covariates did not alter the diagnostic significance of TIMP-1. In conclusion, after analyzing CRC and healthy controls, our data suggest an upper moderate value of TIMP-1 for CRC detection. In detail, pooled analysis of the studies illustrated a relatively specific role for TIMP-1 in predicting CRC, with combined 0.65 sensitivity and 0.87 specificity. The DOR estimate revealed a superior diagnostic accuracy for diagnosing CRC, with a value of 12.73. Furthermore, SROC results showed that TIMP-1 yielded an AUC of 0.77, suggesting that the efficiency of TIMP-1 for CRC diagnosis was considerable. TIMP-1 also performed well in clinical utility when we used likelihood ratios to simulate the clinical scenario.

There are some limitations to this meta-analysis. First, the included studies did not provide the interval period between pathology and TIMP-1 measurement. Despite the possibility of ignoring deviation in the detection results using the QUADAS-2 quality assessment, the right adoption time practically influences the success of TIMP-1 diagnosis. Second, due to the small number of included studies, we only focused on the diagnostic value of TIMP-1 between CRC and healthy controls, though some other studies have raised concerns about the role of TIMP-1 in patients with colorectal adenoma [39–40]. Third, we failed to perform meta-regression and subgroup analyses by age, which may be a potential source of heterogeneity. Finally, only 4 studies directly provided the cutoff value of TIMP-1 concentration for CRC detection, and we did not obtain the diagnostic value of TIMP-1 at different concentrations, which obviously impedes the clinical utility of TIMP-1 measurement. Thus, more detailed studies with larger cohorts of patients are needed to further explore the role of TIMP-1 in different stages of CRC.

## Conclusions

In conclusion, our study demonstrates that TIMP-1 has potential diagnostic value with upper moderate sensitivity and specificity. TIMP-1 measurement might be useful as a noninvasive screening tool for the clinical practice of CRC. More studies are needed to assess the diagnostic value of TIMP-1 in the early stages of CRC.

## Supporting information

S1 Table PRISMA checklist. (DOCX)

S2 Table Meta-analysis-on-genetic-association-studies-form. (DOCX)
